# Changes in dynamic and static brain fluctuation distinguish minimal hepatic encephalopathy and cirrhosis patients and predict the severity of liver damage

**DOI:** 10.3389/fnins.2023.1077808

**Published:** 2023-03-28

**Authors:** Jiang Ji, Yi-yang Liu, Guo-Wei Wu, Yan-Long Hu, Chang-Hua Liang, Xiao-dong Wang

**Affiliations:** ^1^Department of Radiology, General Hospital of Ningxia Medical University, Yinchuan, China; ^2^Department of Radiology, The First Affiliated Hospital of Xinxiang Medical College, Xinxiang, China; ^3^Department of Radiology, The First Affiliated Hospital of Zhengzhou University, Zhengzhou, China; ^4^Chinese Institute for Brain Research, Beijing, China

**Keywords:** minimal hepatic encephalopathy (MHE), dynamic amplitude of low-frequency fluctuations, support vector machine background, amplitude of low-frequency fluctuation (ALFF), support vector machine (SVM)

## Abstract

**Purpose:**

Minimal hepatic encephalopathy (MHE) is characterized by mild neuropsychological and neurophysiological alterations that are not detectable by routine clinical examination. Abnormal brain activity (in terms of the amplitude of low-frequency fluctuation (ALFF) has been observed in MHE patients. However, little is known concerning temporal dynamics of intrinsic brain activity. The present study aimed to investigate the abnormal dynamics of brain activity (dynamic ALFF; dALFF) and static measures [static ALFF; (sALFF)] in MHE patients and to strive for a reliable imaging neuromarkers for distinguishing MHE patients from cirrhosis patients. In addition, the present study also investigated whether intrinsic brain activity predicted the severity of liver damage.

**Methods:**

Thirty-four cirrhosis patients with MHE, 28 cirrhosis patients without MHE, and 33 age-, sex-, and education-matched healthy controls (HCs) underwent resting-state magnetic resonance imaging (rs-fMRI). dALFF was estimated by combining the ALFF method with the sliding-window method, in which temporal variability was quantized over the whole-scan timepoints and then compared among the three groups. Additionally, dALFF, sALFF and both two features were utilized as classification features in a support vector machine (SVM) to distinguish MHE patients from cirrhosis patients. The severity of liver damage was reflected by the Child–Pugh score. dALFF, sALFF and both two features were used to predict Child–Pugh scores in MHE patients using a general linear model.

**Results:**

Compared with HCs, MHE patients showed significantly increased dALFF in the left inferior occipital gyrus, right middle occipital gyrus, and right insula; increased dALFF was also observed in the right posterior lobe of the cerebellum (CPL) and right thalamus. Compared with HCs, noMHE patients exhibited decreased dALFF in the right precuneus. In contrast, compared with noMHE patients, MHE patients showed increased dALFF in the right precuneus, right superior frontal gyrus, and right superior occipital gyrus. Furthermore, the increased dALFF values in the left precuneus were positively associated with poor digit-symbol test (DST) scores (r = 0.356, *p* = 0.038); however, dALFF in the right inferior temporal gyrus (ITG) was negatively associated with the number connection test–A (NCT-A) scores (r = -0.784, *p* = 0.000). A significant positive correlation was found between dALFF in the left inferior occipital gyrus (IOG) and high blood ammonia levels (r = 0.424, *p* = 0.012). Notably, dALFF values yielded a higher classification accuracy than sALFF values in distinguishing MHE patients from cirrhosis patients. Importantly, the dALFF values predicted the Child–Pugh score (r = 0.140, *p* = 0.030), whereas sALFF values did not in the current dataset. Combining two features had high accuracy in classification in distinguishing MHE patients from cirrhotic patients and yielded prediction in the severity of liver damage.

**Conclusion:**

These findings suggest that combining dALFF and sALFF features is a useful neuromarkers for distinguishing MHE patients from cirrhosis patients and highlights the important role of dALFF feature in predicting the severity of liver damage in MHE.

## Introduction

Minimal hepatic encephalopathy (MHE) is considered a subclinical or early stage of hepatic encephalopathy, manifesting with mild abnormalities in cognitive function, neurophysiology, and metabolism (Weissenborn, 2019). Patients with MHE secondary to cirrhosis have a high incidence and have a risk of progression to overt hepatic encephalopathy (OHE), potentially risking individual health and imposing a burden on health care services (Weissenborn et al., 2004; Dhiman et al., 2010; Labenz et al., 2019; Zhang et al., 2020). Notably, neurocognitive dysfunction in MHE patients is subtle and cannot be found by routine clinical exams (Dhiman et al., 2010). Therefore, it is worth investigating the neuropathophysiological mechanism and reliable diagnostic biomarkers of MHE.

Resting-state functional magnetic resonance imaging (rs-fMRI) studies have shown that abnormal intrinsic brain activity can reveal MHE-related biological mechanism. The amplitude of low-frequency fluctuations (ALFF), as a highly sensitive brain measure reflecting intrinsic brain activity, can be used to explore possible mechanisms (Zang et al., 2007). ALFF has been one of the most widely used neuroimaging biomarkers in assessing spontaneous fluctuations in brain activity, which may reflect baseline brain activity underlying disease status (Fryer et al., 2015). For instance, Chen et al. (2016a) confirmed that patients with MHE have abnormal ALFF in multiple brain regions, revealing abnormal baseline spontaneous brain fluctuations related to the neuropathophysiological mechanism. In addition, some researchers argue that abnormal ALFF in certain brain regions may neurophysiological mechanisms, for example, the precuneus, thalamus (Chen et al., 2020a) and prefrontal cortex (Zhong et al., 2016). Several researchers using ALFF as a biological marker found that MHE-related neurophysiological defects were associated with dysfunctions in brain regions belonging to specific subnetworks, e.g., the default-mode network (DMN) and visual network (VN) (Qi et al., 2012).

Many rs-fMRI studies treated brain activity during the scan as a static and uniform process, but increasing studies have shown that spontaneous brain fluctuations are temporally variable (Felipo et al., 2013; Lu et al., 2020; Sun et al., 2021). Combining ALFF with sliding-window approaches, the dynamic ALFF (dALFF) method was proposed to capture the dynamic changes in brain fluctuations over time (Wen et al., 2021), which has been used to study neuropsychiatric diseases, such as drug addiction (Wen et al., 2021), schizophrenia (Fu et al., 2018) and Parkinson’s disease (Zhang et al., 2021). Static ALFF (sALFF) is not a new indicator of intrinsic brain activity compared to dALFF. The sALFF treats ALFF as static throughout all the time frame. Briefly, sALFF indicates the stable activation of brain regions and reflects baseline energy consumption to support basic brain function, while dALFF displays the adaptability and flexibility of spontaneous brain activity through changes in resting-state energy expenditure (Jiang et al., 2020). Therefore, new insights may be gained by using dALFF to explore brain function in MHE. Specifically, we compared the discriminative power of dALFF, sALFF and both two features by using SVM, a typical machine learning classification tool (Chen et al., 2016a; Chen et al., 2020b; Cheng et al., 2021). We also used a general linear model in MHE to predict the Child–Pugh score, a scale commonly used clinically to measure the degree of liver damage in cirrhosis patients.

This research aimed to characterize MHE intrinsic brain activity patterns compared with HCs and cirrhosis without MHE (noMHE). We sought to seek an accurate biomarker to distinguish MHE patients from cirrhosis patients and predict the severity of liver damage.

## Materials and methods

### Participants

The study was approved by the Ethics Committee of Ningxia Medical University General Hospital. Written informed consent was obtained from all subjects before the study. A total of 71 patients diagnosed with hepatitis B virus-related hepatic cirrhosis by clinical evaluations and laboratory examinations were recruited from our Department of Infectious Diseases. Three patients with liver cancer were excluded. The gender-, age-, and education –matched healthy controls (HCs) were recruited from the local community through advertisements. All participants were between 35 and 55 years old, and had 6–11 years of education. Exclusion criteria for patients were (1) any brain disease, neuropsychiatric disorders or related history; (2) history of psychotropic drug addiction; (3) diabetes, anemia or other chronic metabolic conditions; (4) claustrophobia and other contraindications to MRI; (5) inability to complete the Psychometric Hepatic Encephalopathy Score (PHES) examination; and (6) excessive head movement during imaging (translation > *2* mm, rotation >2°). Six patients (four patients with MHE, two patients without MHE) were excluded due to excessive head movements during the scan. In the HC groups, two subjects were excluded due to excessive head movements. Consequently, 62 patients (34 MHE patients, 28 patients without MHE) and 33 HCs were included in the final analyses.

### Neuropsychological assessments

According to previous studies, the diagnosis of mild hepatic encephalopathy is based on neuropsychological assessments from the PHES, which include the type A number connection test (NCT-A) and digit-symbol test (DST) (Cheng et al., 2021). If both of above tests were positive, the patient was diagnosed with mild hepatic encephalopathy, and if one test was positive, the patient was diagnosed with simple cirrhosis. All subjects were evaluated on the NCT-A and DST scales under the guidance of the same specially trained physician before the 1 h BOLD-fMRI scan.

### Laboratory parameters

Before MRI scanning, the following laboratory parameters were measured: venous blood ammonia levels, albumin levels, total bilirubin levels, prothrombin time, and presence of ascites. One day before venous blood sampling, the patient was instructed to avoid smoking, and the sample was placed in a freezer immediately after collection. The Child–Pugh score is a clinically used grading scale for quantitative assessment of liver reserve function in patients with cirrhosis. Higher scores indicate more severe liver damage (Lv et al., 2021). The Child Pugh score includes five different indicators (general condition, venous blood ammonia levels, albumin levels, total bilirubin levels, prothrombin time, and presence of ascites) scored as 1, 2, or 3. Scores on the five indicators are summed, resulting in a minimum score of five and a maximum score of 15. According to the sum, liver reserve function is classified as A (5–6 points), B (7–9 points), or C (10–15 points).

### MRI data acquisition

The scanning device was a GE 3.0T HDMR (SIGNA EXCITE 3.0T HDMR) with an 8-channel head coil. Resting-state BOLD-fMRI date was acquired using a gradient-recalled echo echo-planar imaging (GRE-EPI) sequence with the following parameters: TR/TE = 2,000 ms/30 ms, flip angle = 90^°^, FOV = 240 × 240 mm^2^, matrix = 64 × 64, slice thickness = 3 mm, 35 contiguous axial slices with no slice gap, and volumes = 240. All participants were asked to avoid purposeful thinking and keep their eyes closed.

### Data preprocessing

The Data Processing & Analysis for Brain Imaging (DPABI, v2.3)^1^ Toolkit with Statistical Parametric Mapping (SPM) software (Zang et al., 2007) was used for functional raw data preprocessing. The first 10 time points were removed. The remaining 230 volumes were corrected by slice-timing and realignment for head motion correction (subjects were excluded if they had a maximal head motion displacement >2 mm or rotation >2.0^°^). The mean framewise displacement (FD) of each subject was computed. After spatial normalization to the standard EPI template, the functional dates were resampled to 3 × 3 × 3 mm isotropic voxels. Then, using a linear regression analysis, some spurious variances, including 24 head motion parameters, global signals, white matter signals, and cerebrospinal fluid signals, were regressed out. To ensure consistency across the whole period, the present study did not perform scrubbing (Yan et al., 2013). To reduce motion-related impacts, the group-level analysis was performed with mean FD as a covariate (Supplementary Figure 4). Next, a 6 mm full-width at half-maximum Gaussian kernel was used to smooth the functional maps. Bandpass filters were applied to functional images after linear trends were removed.

### Dynamic and static ALFF calculation

Dynamic ALFF was calculated using the sliding window method in the Dynamic Brain Connectome (Dynamic BC) toolbox (Liao et al., 2014). In this study, each window size included 50 time frames. The window size, as an important parameter, still has no standard configuration. In this study, a window size of 50 time frames (100 s) and a window overlap of 90% [step size of 5 time frames (10 s)] were set to calculate the dALFF values of each subject, as in most previous studies (Cui et al., 2020; Zheng et al., 2021). The whole time series of each subject was divided into 39 time windows. Each window had a corresponding dALFF mapping. We assessed the temporal variability of intrinsic brain activity by calculating the variance of the dynamic brain activity under the 39 time windows. Finally, the dALFF mapping were normalized to z scores for statistical analysis. In addition, to determine whether dALFF provided overlapping or complementary information, we also calculated the sALFF values for each subject by DPABI (Zang et al., 2007). After preprocessing, the time series for each voxel was bandpass filtered (0.01–0.08 Hz) to remove the effects of very-low-frequency drift and high frequency noise. Next, the filtered time series was transformed to a frequency mapping Y with a fast Fourier transform. Then, ALFF was calculated according to the following formula:


$$\text{ALFF}=\,\sum_{\text{i}={\text{N}}_{1}}^{{\text{N}}_{2}}{\text{Y}}_{\text{i}}\,/\,\left({\text{N}}_{2}\,-N_{1}\right)$$

Where N_1_ and N_2_ are the data index locations for the lowest and highest frequencies of the selected band corresponding to the discrete frequency spectrum, respectively. In the present study, the frequency band of 0.01–0.08 Hz and 230 time frames were selected to calculate sALFF values for each group, and the sALFF value of each voxel was divided by the global mean of the sALFF values.

### Classification analysis and Child–Pugh score prediction analysis

The classification performance of abnormal dALFF, sALFF and both two features values at the individual level by SVM based on MVPA for Neuroimaging (MVPANI) software (Peng et al., 2020) distinguished MHE patients from noMHE patients with the following steps. (1) The dALFF, sALFF and both two features mapping of MHE and noMHE patients served as classification features for the SVM method. (2) K-fold cross-validation was utilized to evaluate the SVM model performance. In each trial, all patients (34 MHE and 28 noMHE) were randomly divided into K groups. One of the training folds was selected as the testing dataset. The remaining K-1 groups were used as the training set. Use the selected training dataset to train the model and evaluate it with the testing dataset. K is usually set as 10. (3) To estimate whether the classification results of the classifier model were robust and reliable, this present study used permutations = 5,000 to assess the statistical significance of the model. The total accuracy/specificity/sensitivity metric was used to evaluate the classification performance of the SVM model.

To examine the relationship between changes in intrinsic brain activity and the severity of liver damage, we used a general linear model to predict the Child–Pugh score for each patient in the MHE group. Firstly, we used the different dALFF values of the MHE group and the noMHE group as well as leave-out cross-validation (LOOCV) to produce a robust prediction model. In each LOOCV, we selected one subject’s data as the test set, and the remaining subjects’ data were used as the training set to predict the Child–Pugh score of the subject was predicted based on constructed prediction model. Finally, we used Pearson’s correlation analysis to determine whether the predicted Child–Pugh score was correlated with the observed Child–Pugh score in MHE. In addition, we also employed a LOOCV procedure to predict Child–Pugh scores according to sALFF values and combination of dALFF values and sALFF values.

### Statistical analysis

Clinical and demographics characteristics were evaluated among the three groups by in SPSS (version 23.0). Sex differences were analysed by using the chi-square (χ^2^) test. Differences in, age, years of education and neuropsychological scores among the three groups were assessed by one-way analysis of variance (ANOVA). A two-sample *t*-test in SPM was employed to determine the brain regions with differences in dALFF and sALFF values among the three groups under a gray matter mask. Furthermore, the Spearman correlation analysis was applied to assess the associations of the dALFF and sALFF values in specific brain regions with neuropsychological test scores and blood ammonia levels in the MHE group. Gaussian random field theory (GRF) correction with *P*_*voxel*_ < 0.005 and *P*_*cluster*_ < 0.05 was applied. Pearson’s correlation was employed to determine whether predicted Child–Pugh score is correlated with the observed Child–Pugh score in patients with MHE.

## Results

### Clinical characteristics

The current study included 34 MHE patients, 28 noMHE patients, and 33 HCs. The detailed characteristics and clinical data are displayed in Table 1. Age, sex, and educational differences between the three groups were non-significant. Patients with MHE performed significantly worse than patients without MHE and HCs on cognitive tests.

**TABLE 1:** Comparison of clinical data among subjects in the two groups.

	MHE (*n* = 34)	noMHE (*n* = 28)	HC (*n* = 33)	χ 2/t/F value	*P*-value
Sex (M/F)	19/15	15/13	17/16	0.128^†^	0.937
Age (years)	43.51 ± 7.36	45.32 ± 8.17	46.87 ± 7.24	1.655^‡^	0.197
Education (years)	11.00 ± 2.79	12.12 ± 2.98	11.15 ± 2.78	1.359^‡^	0.262
Child-Pugh score (A/B/C)	2/8/24	1/17/10	–	–	–
NCT-A (seconds)	70.60 ± 35.47	34.24 ± 5.15	20.65 ± 8.43	7.876^§^ 7.430^ǁ^ 5.370^¶^	<0.001^§^ < 0.001^ǁ^ < 0.001^¶^
DST (score)	26.03 ± 20.35	44.86 ± 9.05	44.05 ± 11.00	4.489^§^ 0.310^ǁ^ 4.536^¶^s	<0.001^§^ 0.757^ǁ^ < 0.001^¶^
Ammonia (mg/dl)	71.72 ± 8.5	55.2 ± 6.53	–	6.894	<0.001
Albumin (mg/dl)	42.15 ± 7.52	41.22 ± 7.98	–	0.471	0.640
Total bilirubin (mg/dl)	110.54 ± 86.56	88.56 ± 111.27	–	0.875	0.381
Prothrombin time (seconds)	20.61 ± 6.25	18.83 ± 7.32	–	1.050	0.298
Ascites (no/small/moderate/massive)	2/9/15/5	5/17/5/1	–	–	–

Data are presented as the mean ± standard deviation. ^†^Pearson χ2 test of two groups (two-tailed). ^‡^One-way analysis of variance test among three groups. ^§^Two-sample t-test between the MHE and HC groups (two-tailed). ^ǁ^Two-sample t-test between noMHE and HC groups (two-tailed). ^¶^Two-sample t-test between the MHE and noMHE groups (two-tailed). DST, digit-symbol test; HC, healthy control; MHE, mild hepatic encephalopathy; NCT-A, number connection test of type–A; noMHE, cirrhosis patients without mild hepatic encephalopathy.

### Dynamic and static ALFF among the three groups

**FIGURE 1:**
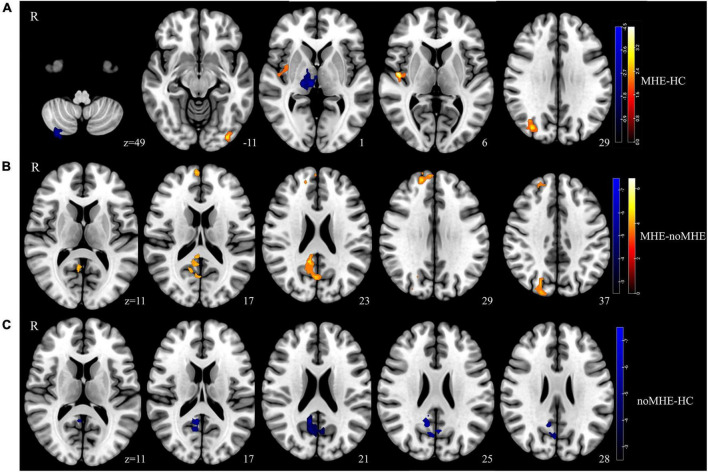
Group differences in the temporal variability of intrinsic brain activity. **(A)** Temporal variability of the dynamic amplitude of low-frequency fluctuation (dALFF) between the minimal hepatic encephalopathy (MHE) and healthy control (HC) groups was identified using two-sample *t*-tests. **(B)** Significance of dALFF values differences between the MHE and noMHE groups. **(C)** Significance of dALFF differences between the cirrhosis without MHE (noMHE) and HC groups. The statistical significance level was set at *P*voxel < 0.005, and *P*cluster < 0.05 [Gaussian random field (GRF)-corrected, cluster extent threshold at k ≥ 40]. Hot colourss represented increased dALFF, and blue colors represent decreased dALFF.

Compared with HCs, MHE patients exhibited reduced dALFF variability mainly in the right CPL and right thalamus, and increased dALFF variability in the right insula, IOG and right middle occipital gyrus (MOG). In contrast, compared with noMHE patients, MHE patients showed increased dALFF in the right precuneus, right superior frontal gyrus (SFG), and right superior occipital gyrus (SOG). noMHE patients showed decreased dALFF in the right precuneus compared with HCs (GRF corrected, cluster size ≥ 40 voxels, voxel-level *p* < 0.005, cluster-level *p* < 0. 05; Figure 1 and Table 2). The present study also found that no or fewer alterations in sALFF. Differences in sALFF among the three groups are shown in the attached Supplementary Data (Supplementary Figure 1 and Supplementary Table 1).

**TABLE 2:** Group differences in dynamic amplitude of low-frequency fluctuation (dALFF) between mild hepatic encephalopathy (MHE) patients, cirrhosis without MHE (noMHE) patients, and healthy controls (HCs).

Group	Brain regions	Brodmann area	MNI coordinates			Cluster size (voxels)	*T* value
			*x*	*y*	*z*		
MHE-HC	Right cerebellum posterior lobe	–	36	–78	–54	55	–4.16
	Right thalamus	–	6	–21	0	54	–4
	Left inferior occipital gyrus	19	–39	–78	–6	44	2.98
	Right middle occipital gyrus	19	33	–75	33	40	3.11
	Right insula	48	48	–9	6	47	4.11
MHE-noMHE	Right precuneus	23	12	–48	24	108	5.07
	Right superior frontal gyrus	32	15	48	21	53	5.11
	Right superior occipital gyrus	19	21	–93	33	50	4.99
noMHE-HC	Right precuneus	23	9	–51	21	92	–6.62

MNI, Montreal Neurological Institute.

### Correlation analyses

**FIGURE 2:**
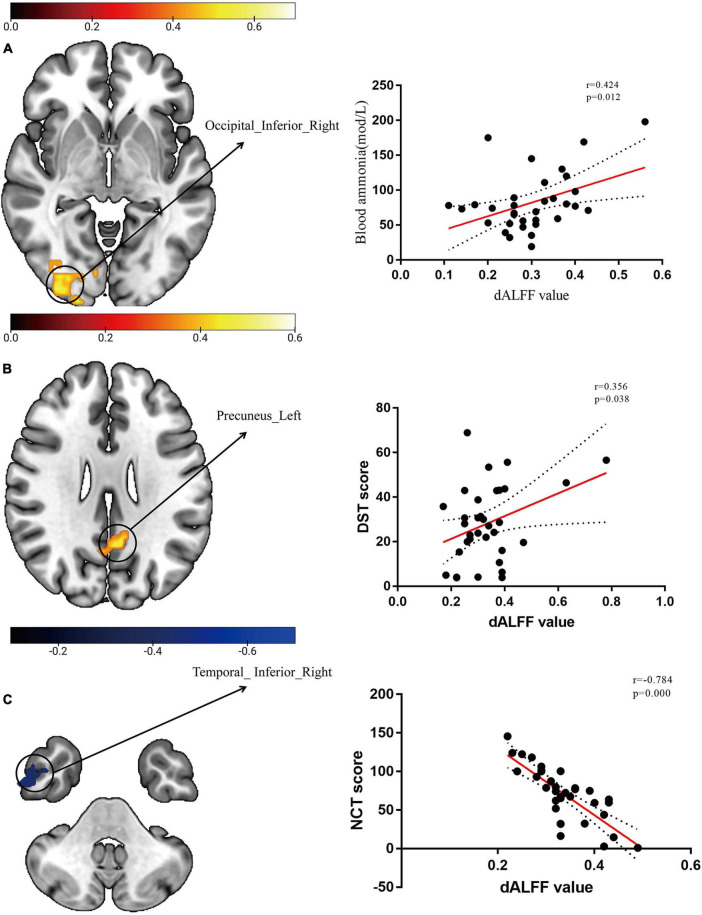
Scatter plots of dynamic amplitude of low-frequency fluctuation (dALFF) with blood ammonia levels and neuropsychological scores. **(A)** The temporal variability of dALFF in the right IOG was positively correlated with blood ammonia levels (r = 0.424, *p* = 0.012, uncorrected). **(B)** The temporal variability of dALFF in the left precuneus gyrus was positively correlated with digit-symbol test (DST) scores (r = 0.356, *p* = 0.038, uncorrected). **(C)** The temporal variability of dALFF in the right inferior temporal gyrus (ITG) was negatively correlated with number connection test–A (NCT-A) scores (r = -0.784, *p* = 0.000, uncorrected).

We extracted dALFF mapping values from the three groups and then performed further correlation analysis with blood ammonia levels and neuropsychological scores. dALFF values in the right IOG was positively correlated with blood ammonia (r = 0.424, *p* = 0.012). Aberrant dALFF values in the left precuneus gyrus were positively correlated with DST scores (r = 0.356, *p* = 0.038). Aberrant dALFF values in the right ITG were negatively correlated with NCT-A scores (r = -0.784, *p* = 0.000). We set a significance threshold of *p* < 0.01 for all correlation analyses (uncorrected; Figure 2). The results of correlation analyses on sALFF patterns are shown in the Supplementary Figure 2. None of the sALFF results were significant.

### Classification performance and Child–Pugh score prediction

**FIGURE 3:**
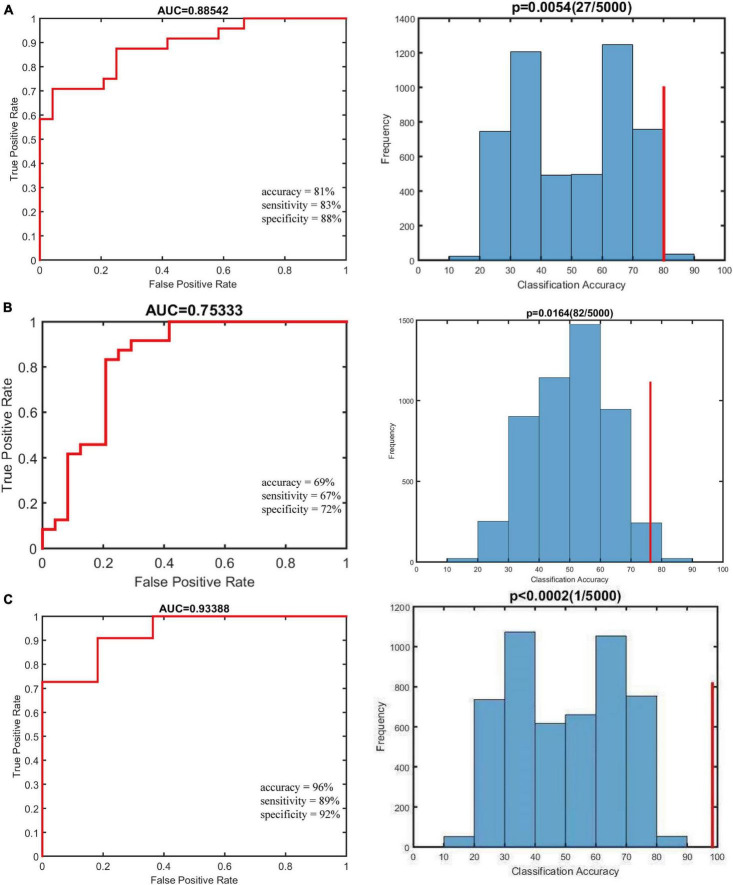
Results of classification analyses. Null distribution on the permutation test (indicated by the histogram) and the actual classification accuracy (indicated by the red vertical line). The *P*-value is shown at the top of the life figure. **(A)** The classification using altered dynamic amplitude of low-frequency fluctuation (dALFF) variability achieved an accuracy of 81%, sensitivity of 83%, and specificity of 88%. **(B)** The classification using altered static amplitude of low-frequency fluctuation (sALFF) achieved an accuracy of 69%, sensitivity of 67%, and specificity of 72%. **(C)** The classification using combination of two features achieved an accuracy of 96%, sensitivity of 89%, specificity of 92%, and the area under curve (AUC) of classification was 0.93.

**FIGURE 4:**
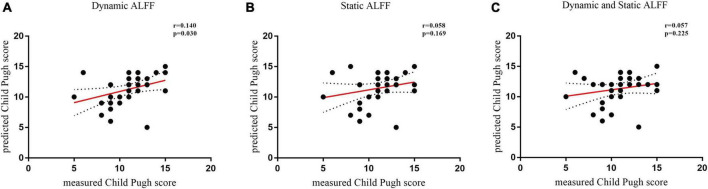
Temporal variability of dynamic amplitude of low-frequency fluctuation (dALFF) predicts Child–Pugh scores. **(A)** The use of dynamic ALFF to predict the Child–Pugh scores (r = 0.140, *p* = 0.030). **(B)** The use of static amplitude of low-frequency fluctuation (sALFF) to predict the Child–Pugh score (r = 0.058, *p* = 0.169). **(C)** The use of combination of dALFF and sALFF features to predict the Child–Pugh score (r = 0.057, *p* = 0.225). Filled circles represent data included in this correlation analysis, while open circles indicate data excluded from this analysis. Solid and dashed red lines represent the best-fit line and 95% confidence intervals of Pearson’s correlation analysis, respectively. ALFF, amplitude of low-frequency fluctuation.

Using individual dALFF values as features, the SVM classification performance reached a total accuracy of 81%, sensitivity of 83%, and specificity of 88%; and the area under curve (AUC) of the classification achieved 0.88. For sALFF, the total accuracy was 69%, the sensitivity was 67%, the specificity was 72%, and the AUC of classification was 0.75. However, for the combination of two features, the total accuracy was 96%, the sensitivity was 89%, the specificity was 92%, and the AUC of classification was 0.93. Thus, dALFF was more useful for classification than sALFF, and the combination of two features play a higher accuracy classification performance than single dALFF or sALFF features. Permutation tests for the three classification analyses showed *p* < 0.001 (Figure 3). We also found that dALFF values predicted the Child–Pugh scores (r = 0.140, *p* = 0.030), while sALFF values and combination of dALFF values and sALFF values did not (r = 0.058, *p* = 0.169; r = 0.057, *p* = 0.225) (Figure 4).

### Validation analysis

We chose 30 time frames and 80 time frames to recalculate the primary results to confirm the impact of dALFF variability on the outcomes at various sliding-window lengths. See (Supplementary Figure 3) for detailed validation results.

## Discussion

This study used dALFF and sALFF to investigate brain fluctuation characteristics in cirrhosis patients with and without MHE as well as their relationships with cognitive dysfunction. Anthors found d, dALFF showed similar and complementary brain activation information compared with the sALFF. The classification accuracy was superior when using combination of dALFF and sALFF compared with that using dALFF as features or sALFF as features. Additionally, the altered dALFF values in these regions between the MHE group and the noMHE group could predict the severity of liver damage.

The dynamic characteristics of brain activity, commonly measured by dALFF, reflect intrinsic brain function during mental and cognitive processes (Wang et al., 2016; Kucyi et al., 2017) and have recently been widely used as neuroimaging markers to deepen knowledge about the neural mechanisms underlying various neurological and psychiatric disorders (Kim et al., 2017; Li et al., 2018). Although the cognitive deficits in MHE were confirmed to be associated with abnormal dynamic functional connectivity networks (Chen et al., 2017; Cheng et al., 2021; Cai et al., 2022), whether the time-varying patterns of regional intrinsic brain activity are aberrant remains unknown. Compared to dynamic functional connectivity, dynamic brain activity can be presented with respect to captures fluctuation in spatial variability and strength using first-level statistics (Fu et al., 2018). The present study extends the findings regarding altered dynamic functional connectivity in patients with cirrhosis and characterizes the time-varying patterns of intrinsic regional brain activity in patients with cirrhosis.

### Three classification models for distinguishing MHE

Our study proposes the combination of dALFF and sALFF as a reliable imaging neuromarkers for the detection of MHE from noMHE. noMHE has the potential to progress into MHE, which can cause impairment of cognitive function and further progress to irreversible brain damage (Tapper et al., 2020). Therefor it is important to distinguish MHE from noMHE. SVM is a classifier with high classification accuracy that solves the classification problem of small sample sizes that are non-linear and have high dimensionality (Peng et al., 2020). Some researchers have also applied SVM classifiers to help diagnose MHE patients. For example, Chen et al. used an SVM classifier to distinguish MHE patients from HCs, identifying six brain regions with sALFF values with the best discriminative power (Chen et al., 2016a). Others found that a combination of the SVM approach and regional homogeneity (ReHo) helped to identify MHE patients (Chen et al., 2016b). Chen et al. confirmed that SVM analysis based on SVM analysis based on GM volumetry has the potential to help diagnose MHE in cirrhotic patients (Chen et al., 2020b). In addition, someone confirmed that compared with static features, dynamic features were better for distinguishing MHE patients from HCs (Cheng et al., 2021). In this present study anthors set three classification models (dALFF, sALFF and both two features) by SVM to compare their classification performance in distinguish MHE patients from noMHE. In this study, anthors achieved a high accuracy of 81% for the classification of dALFF values as a feature, which improved by 12% accuracy than sALFF values as a feature. In addition, comparing combining two features model with single feature model, the former obviously improved classification accuracies when comparing single dALFF model and sALFF model by 15, and 27%, respectively. These findings suggest that combining models may be a powerful neuroimaging biomarker for the detection of MHE. The underlying reason for this is that this combining two features model not only considered intrinsic brain activity intensity information but also consider the dynamic changes of intrinsic brain activity in MHE. So only by considering the intrinsic activity intensity effects of the brain in space and the time-varying effects in time, can the MHE complex fluctuation information of the brain be accurately characterized.

The presence of the null distribution of the permutation test is often attributed to the overall number of classification groups and the SVM weights of the two features (Gaonkar et al., 2015; Linn et al., 2016; Krell et al., 2017). In the present study the same numbers in each classification model were used for dALFF and sALFF, and in the dALFF permutation test anthors observed a null distribution with a bimodal distribution. Therefore, anthors believe that this bimodal frequency distribution for dALFF is due to the significant difference in the dALFF classification weights between the two classification models. Anthors also combined the use of dALFF and sALFF to distinguish patients from noMHE cirrhosis patients; this method had high accuracy in classification with more significantly different amounts, and yielded a bimodal frequency distribution for dALFF.

### Prediction model for the severity of liver damage

More importantly, dALFF predicted the severity of liver damage. Previous studies have found that neuroimaging features are related to the severity of liver damage (Lin et al., 2019; Ye et al., 2020; Lin et al., 2022). Anthors are not aware of any prior study that has reported employing dynamic values to predict the severity of live damage. Anthors found that dALFF values successfully predicted the severity of liver damage while sALFF values and the combination of dALFF and sALFF did not, which suggest that dALFF values may be a more powerful neuromarker early warning liver damage for MHE in the current sample. Anthors speculated that the poor predictive performance of the combination dALFF and sALFF may be caused by the small sample size. This present study did not underestimate the key role of sALFF in MHE diagnosis. Further studies are needed to verify the contribution of sALFF in MHE identification.

### Altered local brain activity (dALFF and sALFF) in related functional brain regions

Minimal hepatic encephalopathy patients showed increased dALFF variability in both the IOG and MOG and decreased sALFF in bilateral superior occipital gyrus, which are involved in visual information processing (Lee et al., 2000; Wandell et al., 2007). Notably, a decline in visual function is a prominent feature of MHE (Amodio et al., 2004; Arias et al., 2015). The occipital lobe is a key node of the visual network that is involved in visual information integration and processing (Wang et al., 2019). Many fMRI studies have reported incongruous and irregular neuronal connectivity in the visual cortex in MHE patients (Bajaj et al., 2017; Zhang et al., 2017a; Wang et al., 2019). Similar to Cheng et al. (2021)’s results, our findings suggest that the abnormal temporal variability of the occipital lobe is associated with impaired visual processing in MHE patients. Moreover, we found that the increased dALFF value in the left IOG was positively correlated with blood ammonia levels. The degradation of ammonia, a main toxic substance in the brain, the results in increased glutamine levels in astrocytes, causing both swelling and dysfunction of these cells (Córdoba and Mínguez, 2008). Some research has suggested that in MHE patients, the aberrant ALFF in the bilateral cuneus/superior occipital lobule is related to the toxic effects of blood ammonia levels (Chen et al., 2012a; Qi et al., 2012). Thus, its abnormal brain activity in the occipital lobe could result in visual-related dysfunction, which may be caused by the degradation of ammonia.

The present study also found considerably increased dALFF in the right insular cortex in MHE patients. Some studies have found that the insula is involved in executive function in MHE patients (Chen et al., 2012a; Qi et al., 2012). The insula, as a critical hub linking the prefrontal and parietal lobes and complex brain networks, plays an important role in transmitting top-down information (Menon and Uddin, 2010; Woo et al., 2017). Top-down processing may involve activation of areas associated with executive function (Diamond, 2013). In addition, the salience network (SN), including the dorsal anterior cingulate and anterior insular cortices, is a brain network that can regulate resource allocation in other brain networks to adapt to changing environmental conditions, such as by helping to reduce DMN activity and increase processing of external stimuli (Seeley et al., 2007). Alterations in the insular cortex, including reduced network efficiency and aberrant spontaneous activity, may upset the balance of executive function-associated networks, resulting in attention deficits and reduced executive function (Bajaj et al., 2009). Furthermore, the insular cortex, itself a limbic structure, is connected to a set of other limbic and related areas, such as the cingulate gyrus, hippocampus, parahippocampal gyrus, dental nucleus, papillary nuclei and amygdala, whose functions are related to emotion, behavior, working memory and perceptual processing (Vogt et al., 2003; Rolls, 2019). Anthors also observed increased sALFF differences in the left parahippocampal gyrus between MHE patients and HCs. Therefore, our study suggests that aberrant dALFF in the right insula may underlie the abnormal executive function, emotional or behavioral regulation, learning and memory performance of MHE patients as well as the interconnection of the above information.

The functions of the thalamus and cerebellum are closely related to executive control and emotional regulation (Bostan and Strick, 2018; Dacre et al., 2021) and are involved in the initial discrimination of emotional and sensory information (Cummings, 1993; Smith et al., 2002; Philp et al., 2014). Anthors found considerably lower dALFF in the right thalamus and CPL and higher sALFF in the right thalamus in MHE patients. Temporal variability in brain fluctuations, such as increased (decreased) dALFF, indicates weakened (strengthened) stability (Christoff et al., 2016), may be the result of changes or adaptation in cognitive function and alterations in pathophysiological states (Preti et al., 2017). Therefore, in light of previous studies, anthors speculate that aberrant thalamic and cerebellar structure and function may contribute to cognitive impairments in MHE. In addition, cerebellar and cerebral cortical function are not isolated but rather were connected by important circuits, such as the corticocerebellar–thalamic–cortical circuit (CCTCC), which regulates neurobehavioral and executive functions (Castellazzi et al., 2018; Li et al., 2022). Therefore, anthors believe that disturbed synchronization of neural activity in the right thalamus and CPL may disturb the balance of the CCTCC. Importantly, in this study, anthors found that the right thalamus indicated by two different brain fluctuation detection methods. These results were in line with earlier static ALFF investigations that found that cirrhotic patients with and without MHE had abnormal local brain functional activity in the thalamus (Zhang et al., 2017a; Li et al., 2019; Zhang et al., 2020). Most likely, the thalamus may be a critical pathological node for understanding the neurophysiological mechanisms of MHE, and hypoactivation of the thalamus can reveal the mechanisms underlying cognitive impairment in MHE.

Increased dALFF values in the right SFG and right precuneus were found in MHE patients compared with those noMHE groups. The frontal lobe, as the most powerful functional region of the brain, participates in spatial working memory and information processing; the prefrontal lobe also supports executive function and emotion regulation (Funahashi, 2006). For MHE patients, abnormalities in the SFG may disrupt visuospatial functioning (Liao et al., 2012; Zhang et al., 2017b). The precuneus participates in visuospatial integration and working memory, deficits in which are associated with MHE (Chen et al., 2012a; Ni et al., 2012; Chen et al., 2013). Therefore, anthors hypothesize that aberrant neural activity in the precuneus contributes to impaired visual function and impaired recall of specific memories in MHE patients. Consistent with this notion, scores on the DST score, one of the neuropsychological assessments that may reflects psychomotor, visuomotor, attention, speed, and visual memory impairment, was significantly positively correlated with the dALFF values in the precuneus in this study. Together with previous studies, this finding shows that the precuneus may be more important than changes in other brain regions in understanding the biological mechanisms underlying cirrhosis-related cognitive dysfunction (Chen et al., 2012b; Ni et al., 2014; Cheng et al., 2017). In addition, the inferior frontal gyrus and precuneus are crucial hubs of the DMN (Chen et al., 2017). Previous studies have shown that the DMN is impaired to varying degrees in MHE patients (Qi et al., 2012; Tsai et al., 2019). According to previous and current finding, anthors propose that abnormal brain activity in the DMN may be a non-invasive and accurate neuroimaging biomarker for identifying patients with MHE.

Earlier investigations have reported altered precuneus activity in patients with noMHE (Ni et al., 2012; Cheng et al., 2017; Zhang et al., 2017b). Consistent with prior studies, anthors discovered reduced dALFF in the precuneus in cirrhosis patients without MHE compared to that in HCs. The precuneus may participate in short-term memory and enhance the attention-modulated visual field (Halbertsma et al., 2020; Luo et al., 2020). As a result, anthors suggest that reduced dALFF in the precuneus disrupts the balance of visual information regulation and results in various cognitive disorders connected to vision, such as deficiencies in visual memory, visuomotor function, and visuospatial thinking. sALFF did not reveal significantly differ between the noMHE patients and HCs.

This study has some limitations. First, the sample size was relatively small. Larger samples are required in the future to confirm our findings. Second, the size of the sliding window remains under discussion. This present study conducted additional experiments (30 time frames and 80 time frames) to verify that the impact of different sliding window sizes on the experimental results; these results differed only slightly. Fifty time frames met the criterion of the minimum length less than 1/f_min_ (52); thus, it is more frequently used in similar studies (Li et al., 2020; Ma et al., 2020; Yang et al., 2022). Third, the reproducibility of predictive models is an unavoidable issue (Woo et al., 2017). In future investigations, the model of MHE brain activity dynamics in the present study should be adapted to include additional participants at various study centers. Compared with leave-one-out cross-validation (LOOCV), K-fold cross-validation has less variance in prediction and is more suitable for studies with small samples (Shen et al., 2017). In addition, SVM is sensitive to differences in feature scales, which have a strong influence on SVM accuracy verification. Finally, anthors did not exclude unavailable time nodes from the scan time series, but anthors used the mean FD as a covariate in statistical analysis to balance the effect of scrubbing bad time points and eliminate the effect of head movement on our experimental results.

## Conclusion

In summary, this study revealed that abnormal dALFF and sALFF in MHE patients mainly affected brain regions or networks associated with visual function, cognitive control and executive function, emotion regulation, and spatial working memory, indicating that reduced or impaired visual function dysfunction, impaired cognition, emotion regulation and spatial memory were associated with MHE. More broadly, combination dALFF and sALFF features play a higher accuracy classification performance than single dALFF or sALFF features., and these dALFF abnormalities predicted the severity of liver damage, while sALFF and combination dALFF and sALFF abnormalities did not. This novel study suggests that combining dALFF and sALFF is better to distinguish MHE from cirrhosis patients and highlights the important contribution of alterations in dALFF variability for predicting the severity of liver damage in MHE patients.

## Data availability statement

The original contributions presented in this study are included in the article/Supplementary material, further inquiries can be directed to the corresponding authors.

## Ethics statement

The studies involving human participants were reviewed and approved by General Hospital of Ningxia Medical University. The patients/participants provided their written informed consent to participate in this study.

## Author contributions

JJ: manuscript preparation, literature research, and data analysis. Y-YL and Y-LH: literature research and data analysis. G-WW: guidance of imaging knowledge and data analysis. C-HL and X-DW: study conception and design, manuscript review, and guarantor of integrity of the entire study. All authors have read and approved the final manuscript.

## Footnotes

1
www.rfmri.org/dpabi

## References

[B1] Amodio P. Montagnese S. Gatta A. Morgan M. Y. (2004). Characteristics of minimal hepatic encephalopathy. *Metab. Brain Dis.* 19 253–267. 10.1023/b:mebr.0000043975.01841.de 15554421

[B2] Arias N. Méndez M. Gómez-Lázaro E. Azpiroz A. Arias J. L. (2015). Main target of minimal hepatic encephalopathy: Morphophysiological, inflammatory and metabolic view. *Physiol. Behav.* 149 247–254. 10.1016/j.physbeh.2015.06.019 26079568

[B3] Bajaj J. S. Ahluwalia V. Thacker L. R. Fagan A. Gavis E. A. Lennon M. (2017). Brain training with video games in covert hepatic encephalopathy. *Am. J. Gastroenterol.* 112 316–324. 10.1038/ajg.2016.544 27958279

[B4] Bajaj J. S. Wade J. B. Sanyal A. J. (2009). Spectrum of neurocognitive impairment in cirrhosis: Implications for the assessment of hepatic encephalopathy. *Hepatology* 50 2014–2021. 10.1002/hep.23216 19787808

[B5] Bostan A. C. Strick P. L. (2018). The basal ganglia and the cerebellum: Nodes in an integrated network. *Nat. Rev. Neurosci.* 19 338–350. 10.1038/s41583-018-0002-7 29643480 PMC6503669

[B6] Cai L. M. Shi J. Y. Dong Q. Y. Wei J. Chen H. J. (2022). Aberrant stability of brain functional architecture in cirrhotic patients with minimal hepatic encephalopathy. *Brain Imaging Behav.* 16 2258–2267. 10.1007/s11682-022-00696-9 35729463

[B7] Castellazzi G. Bruno S. D. Toosy A. T. Casiraghi L. Palesi F. Savini G. (2018). Prominent changes in cerebro-cerebellar functional connectivity during continuous cognitive processing. *Front. Cell. Neurosci.* 12:331. 10.3389/fncel.2018.00331 30327590 PMC6174227

[B8] Chen H. J. Jiao Y. Zhu X. Q. Zhang H. Y. Liu J. C. Wen S. (2013). Brain dysfunction primarily related to previous overt hepatic encephalopathy compared with minimal hepatic encephalopathy: Resting-state functional MR imaging demonstration. *Radiology* 266 261–270. 10.1148/radiol.12120026 23047839

[B9] Chen H. J. Lin H. L. Chen Q. F. Liu P. F. (2017). Altered dynamic functional connectivity in the default mode network in patients with cirrhosis and minimal hepatic encephalopathy. *Neuroradiology* 59 905–914. 10.1007/s00234-017-1881-4 28707166

[B10] Chen H. J. Zhang L. Jiang L. F. Chen Q. F. Li J. Shi H. B. (2016a). Identifying minimal hepatic encephalopathy in cirrhotic patients by measuring spontaneous brain activity. *Metab. Brain Dis.* 31 761–769. 10.1007/s11011-016-9799-9 26886109

[B11] Chen H. J. Zhu X. Q. Jiao Y. Li P. C. Wang Y. Teng G. J. (2012a). Abnormal baseline brain activity in low-grade hepatic encephalopathy: A resting-state fMRI study. *J. Neurol. Sci.* 318 140–145. 10.1016/j.jns.2012.02.019 22541365

[B12] Chen H. J. Zhu X. Q. Yang M. Liu B. Zhang Y. Wang Y. (2012b). Changes in the regional homogeneity of resting-state brain activity in minimal hepatic encephalopathy. *Neurosci. Lett.* 507 5–9. 10.1016/j.neulet.2011.11.033 22178142

[B13] Chen L. H. Shi J. Y. Zou T. X. Zhang L. Gou Y. Lin Y. (2020a). Disturbance of thalamic metabolism and its association with regional neural dysfunction and cognitive impairment in minimal hepatic encephalopathy. *Eur. J. Radiol.* 131:109252. 10.1016/j.ejrad.2020.109252 32949859

[B14] Chen Q. F. Chen H. J. Liu J. Sun T. Shen Q. T. (2016b). Machine learning classification of cirrhotic patients with and without minimal hepatic encephalopathy based on regional homogeneity of intrinsic brain activity. *PLoS One* 11:e0151263. 10.1371/journal.pone.0151263 26978777 PMC4792397

[B15] Chen Q. F. Zou T. X. Yang Z. T. Chen H. J. (2020b). Identification of patients with and without minimal hepatic encephalopathy based on gray matter volumetry using a support vector machine learning algorithm. *Sci. Rep.* 10:2490. 10.1038/s41598-020-59433-1 32051514 PMC7016173

[B16] Cheng Y. Huang L. X. Zhang L. Ma M. Xie S. S. Ji Q. (2017). Longitudinal intrinsic brain activity changes in cirrhotic patients before and one month after liver transplantation. *Korean J. Radiol.* 18 370–377. 10.3348/kjr.2017.18.2.370 28246517 PMC5313525

[B17] Cheng Y. Zhang G. Zhang X. Li Y. Li J. Zhou J. (2021). Identification of minimal hepatic encephalopathy based on dynamic functional connectivity. *Brain Imaging Behav.* 15 2637–2645. 10.1007/s11682-021-00468-x 33755921

[B18] Christoff K. Irving Z. C. Fox K. C. Spreng R. N. Andrews-Hanna J. R. (2016). Mind-wandering as spontaneous thought: A dynamic framework. *Nat. Rev. Neurosci.* 17 718–731. 10.1038/nrn.2016.113 27654862

[B19] Córdoba J. Mínguez B. (2008). Hepatic encephalopathy. *Semin. Liver Dis.* 28 70–80. 10.1055/s-2008-1040322 18293278

[B20] Cui Q. Sheng W. Chen Y. Pang Y. Lu F. Tang Q. (2020). Dynamic changes of amplitude of low-frequency fluctuations in patients with generalized anxiety disorder. *Hum. Brain Mapp.* 41 1667–1676. 10.1002/hbm.24902 31849148 PMC7267950

[B21] Cummings J. L. (1993). Frontal-subcortical circuits and human behavior. *Arch. Neurol.* 50 873–880. 10.1001/archneur.1993.00540080076020 8352676

[B22] Dacre J. Colligan M. Clarke T. Ammer J. J. Schiemann J. Chamosa-Pino V. (2021). A cerebellar-thalamocortical pathway drives behavioral context-dependent movement initiation. *Neuron* 109 2326–2338.e8. 10.1016/j.neuron.2021.05.016 34146469 PMC8315304

[B23] Dhiman R. K. Kurmi R. Thumburu K. K. Venkataramarao S. H. Agarwal R. Duseja A. (2010). Diagnosis and prognostic significance of minimal hepatic encephalopathy in patients with cirrhosis of liver. *Dig. Dis. Sci.* 55 2381–2390. 10.1007/s10620-010-1249-7 20508990

[B24] Diamond A. (2013). Executive functions. *Annu. Rev. Psychol.* 64 135–168. 10.1146/annurev-psych-113011-143750 23020641 PMC4084861

[B25] Felipo V. Urios A. Valero P. Sánchez M. Serra M. A. Pareja I. (2013). Serum nitrotyrosine and psychometric tests as indicators of impaired fitness to drive in cirrhotic patients with minimal hepatic encephalopathy. *Liver Int.* 33 1478–1489. 10.1111/liv.12206 23714168

[B26] Fryer S. L. Roach B. J. Ford J. M. Turner J. A. van Erp T. G. Voyvodic J. (2015). Relating intrinsic low-frequency BOLD cortical oscillations to cognition in schizophrenia. *Neuropsychopharmacology* 40 2705–2714. 10.1038/npp.2015.119 25944410 PMC4864646

[B27] Fu Z. Tu Y. Di X. Du Y. Pearlson G. D. Turner J. A. (2018). Characterizing dynamic amplitude of low-frequency fluctuation and its relationship with dynamic functional connectivity: An application to schizophrenia. *Neuroimage* 180(Pt B), 619–631. 10.1016/j.neuroimage.2017.09.035 28939432 PMC5860934

[B28] Funahashi S. (2006). Prefrontal cortex and working memory processes. *Neuroscience* 139 251–261. 10.1016/j.neuroscience.2005.07.003 16325345

[B29] Gaonkar B. Shinohara R. Davatzikos C. Alzheimers Disease Neuroimaging Initiative (2015). Interpreting support vector machine models for multivariate group wise analysis in neuroimaging. *Med. Image Anal.* 24 190–204. 10.1016/j.media.2015.06.008 26210913 PMC4532600

[B30] Halbertsma H. N. Elshout J. A. Bergsma D. P. Norris D. G. Cornelissen F. W. van den Berg A. V. (2020). Functional connectivity of the Precuneus reflects effectiveness of visual restitution training in chronic hemianopia. *Neuroimage Clin.* 27:102292. 10.1016/j.nicl.2020.102292 32554320 PMC7303670

[B31] Jiang S. F. Shi J. Y. Yang Z. T. Zhang L. Chen H. J. (2020). Aberrant dynamic functional network connectivity in cirrhotic patients without overt hepatic encephalopathy. *Eur. J. Radiol.* 132:109324. 10.1016/j.ejrad.2020.109324 33038576

[B32] Kim J. Criaud M. Cho S. S. Díez-Cirarda M. Mihaescu A. Coakeley S. (2017). Abnormal intrinsic brain functional network dynamics in Parkinson’s disease. *Brain* 140 2955–2967. 10.1093/brain/awx233 29053835 PMC5841202

[B33] Krell M. M. Wilshusen N. Seeland A. Kim S. K. (2017). Classifier transfer with data selection strategies for online support vector machine classification with class imbalance. *J. Neural Eng.* 14:025003. 10.1088/1741-2552/aa5166 28192282

[B34] Kucyi A. Hove M. J. Esterman M. Hutchison R. M. Valera E. M. (2017). Dynamic brain network correlates of spontaneous fluctuations in attention. *Cereb. Cortex* 27 1831–1840. 10.1093/cercor/bhw029 26874182 PMC6317462

[B35] Labenz C. Toenges G. Schattenberg J. M. Nagel M. Sprinzl M. F. Nguyen-Tat M. (2019). Clinical predictors for poor quality of life in patients with covert hepatic encephalopathy. *J. Clin. Gastroenterol.* 53 e303–e307. 10.1097/mcg.0000000000001149 30439761

[B36] Lee H. W. Hong S. B. Seo D. W. Tae W. S. Hong S. C. (2000). Mapping of functional organization in human visual cortex: Electrical cortical stimulation. *Neurology* 54 849–854. 10.1212/wnl.54.4.849 10690975

[B37] Li J. L. Jiang H. Zhang X. D. Huang L. X. Xie S. S. Zhang L. (2019). Microstructural brain abnormalities correlate with neurocognitive dysfunction in minimal hepatic encephalopathy: A diffusion kurtosis imaging study. *Neuroradiology* 61 685–694. 10.1007/s00234-019-02201-4 30918990

[B38] Li Q. Cao X. Liu S. Li Z. Wang Y. Cheng L. (2020). Dynamic alterations of amplitude of low-frequency fluctuations in patients with drug-naïve first-episode early onset schizophrenia. *Front. Neurosci.* 14:901. 10.3389/fnins.2020.00901 33122982 PMC7573348

[B39] Li R. Liao W. Yu Y. Chen H. Guo X. Tang Y. L. (2018). Differential patterns of dynamic functional connectivity variability of striato-cortical circuitry in children with benign epilepsy with centrotemporal spikes. *Hum. Brain Mapp.* 39 1207–1217. 10.1002/hbm.23910 29206330 PMC6866449

[B40] Li Y. Yang L. Li L. Xie Y. Fang P. (2022). The resting-state cerebro-cerebellar function connectivity and associations with verbal working memory performance. *Behav. Brain Res.* 417:113586. 10.1016/j.bbr.2021.113586 34536430

[B41] Liao L. M. Zhou L. X. Le H. B. Yin J. J. Ma S. H. (2012). Spatial working memory dysfunction in minimal hepatic encephalopathy: An ethology and BOLD-fMRI study. *Brain Res.* 1445 62–72. 10.1016/j.brainres.2012.01.036 22325099

[B42] Liao W. Wu G. R. Xu Q. Ji G. J. Zhang Z. Zang Y. F. (2014). DynamicBC: A MATLAB toolbox for dynamic brain connectome analysis. *Brain Connect.* 4 780–790. 10.1089/brain.2014.0253 25083734 PMC4268585

[B43] Lin S. Guo Z. Chen S. Lin X. Ye M. Qiu Y. (2022). Progressive brain structural impairment assessed via network and causal analysis in patients with hepatitis B virus-related cirrhosis. *Front. Neurol.* 13:849571. 10.3389/fneur.2022.849571 35599731 PMC9120530

[B44] Lin W. Chen X. Gao Y. Q. Yang Z. T. Yang W. Chen H. J. (2019). Hippocampal atrophy and functional connectivity disruption in cirrhotic patients with minimal hepatic encephalopathy. *Metab. Brain Dis.* 34 1519–1529. 10.1007/s11011-019-00457-6 31363985

[B45] Linn K. A. Gaonkar B. Satterthwaite T. D. Doshi J. Davatzikos C. Shinohara R. T. (2016). Control-group feature normalization for multivariate pattern analysis of structural MRI data using the support vector machine. *Neuroimage* 132 157–166. 10.1016/j.neuroimage.2016.02.044 26915498 PMC4851898

[B46] Lu F. Liu P. Chen H. Wang M. Xu S. Yuan Z. (2020). More than just statics: Abnormal dynamic amplitude of low-frequency fluctuation in adolescent patients with pure conduct disorder. *J. Psychiatr. Res.* 131 60–68. 10.1016/j.jpsychires.2020.08.027 32937251

[B47] Luo Z. Zeng L. L. Qin J. Hou C. Shen H. Hu D. (2020). Functional parcellation of human brain precuneus using density-based clustering. *Cereb. Cortex* 30 269–282. 10.1093/cercor/bhz086 31044223

[B48] Lv Y. Wang Z. Li K. Wang Q. Bai W. Yuan X. (2021). Risk stratification based on chronic liver failure consortium acute decompensation score in patients with child-pugh B cirrhosis and acute variceal bleeding. *Hepatology* 73 1478–1493. 10.1002/hep.31478 32706906

[B49] Ma M. Zhang H. Liu R. Liu H. Yang X. Yin X. (2020). Static and dynamic changes of amplitude of low-frequency fluctuations in cervical discogenic pain. *Front. Neurosci.* 14:733. 10.3389/fnins.2020.00733 32760245 PMC7372087

[B50] Menon V. Uddin L. Q. (2010). Saliency, switching, attention and control: A network model of insula function. *Brain Struct. Funct.* 214 655–667. 10.1007/s00429-010-0262-0 20512370 PMC2899886

[B51] Ni L. Qi R. Zhang L. J. Zhong J. Zheng G. Wu X. (2014). Brain regional homogeneity changes following transjugular intrahepatic portosystemic shunt in cirrhotic patients support cerebral adaptability theory–a resting-state functional MRI study. *Eur. J. Radiol.* 83 578–583. 10.1016/j.ejrad.2013.10.027 24299611

[B52] Ni L. Qi R. Zhang L. J. Zhong J. Zheng G. Zhang Z. (2012). Altered regional homogeneity in the development of minimal hepatic encephalopathy: A resting-state functional MRI study. *PLoS One* 7:e42016. 10.1371/journal.pone.0042016 22848692 PMC3404989

[B53] Peng Y. Zhang X. Li Y. Su Q. Wang S. Liu F. (2020). MVPANI: A toolkit with friendly graphical user interface for multivariate pattern analysis of neuroimaging data. *Front. Neurosci.* 14:545. 10.3389/fnins.2020.00545 32742251 PMC7364177

[B54] Philp D. J. Korgaonkar M. S. Grieve S. M. (2014). Thalamic volume and thalamo-cortical white matter tracts correlate with motor and verbal memory performance. *Neuroimage* 91 77–83. 10.1016/j.neuroimage.2013.12.057 24401559

[B55] Preti M. G. Bolton T. A. Van De Ville D. (2017). The dynamic functional connectome: State-of-the-art and perspectives. *Neuroimage* 160 41–54. 10.1016/j.neuroimage.2016.12.061 28034766

[B56] Qi R. Zhang L. Wu S. Zhong J. Zhang Z. Zhong Y. (2012). Altered resting-state brain activity at functional MR imaging during the progression of hepatic encephalopathy. *Radiology* 264 187–195. 10.1148/radiol.12111429 22509052

[B57] Rolls E. T. (2019). The cingulate cortex and limbic systems for emotion, action, and memory. *Brain Struct. Funct.* 224 3001–3018. 10.1007/s00429-019-01945-2 31451898 PMC6875144

[B58] Seeley W. W. Menon V. Schatzberg A. F. Keller J. Glover G. H. Kenna H. (2007). Dissociable intrinsic connectivity networks for salience processing and executive control. *J. Neurosci.* 27 2349–2356. 10.1523/jneurosci.5587-06.2007 17329432 PMC2680293

[B59] Shen X. Finn E. S. Scheinost D. Rosenberg M. D. Chun M. M. Papademetris X. (2017). Using connectome-based predictive modeling to predict individual behavior from brain connectivity. *Nat. Protoc.* 12 506–518. 10.1038/nprot.2016.178 28182017 PMC5526681

[B60] Smith K. A. Ploghaus A. Cowen P. J. McCleery J. M. Goodwin G. M. Smith S. (2002). Cerebellar responses during anticipation of noxious stimuli in subjects recovered from depression. Functional magnetic resonance imaging study. *Br. J. Psychiatry* 181 411–415. 10.1192/bjp.181.5.411 12411267

[B61] Sun F. Liu Z. Yang J. Fan Z. Yang J. (2021). Differential dynamical pattern of regional homogeneity in bipolar and unipolar depression: A preliminary resting-state fMRI study. *Front. Psychiatry* 12:764932. 10.3389/fpsyt.2021.764932 34966303 PMC8710770

[B62] Tapper E. B. Zhao L. Nikirk S. Baki J. Parikh N. D. Lok A. S. (2020). Incidence and bedside predictors of the first episode of overt hepatic encephalopathy in patients with cirrhosis. *Am. J. Gastroenterol.* 115 2017–2025. 10.14309/ajg.0000000000000762 32773463 PMC7853725

[B63] Tsai C. F. Tu P. C. Wang Y. P. Chu C. J. Huang Y. H. Lin H. C. (2019). Altered cognitive control network is related to psychometric and biochemical profiles in covert hepatic encephalopathy. *Sci. Rep.* 9:6580. 10.1038/s41598-019-42957-6 31036843 PMC6488566

[B64] Vogt B. A. Berger G. R. Derbyshire S. W. (2003). Structural and functional dichotomy of human midcingulate cortex. *Eur. J. Neurosci.* 18 3134–3144. 10.1111/j.1460-9568.2003.03034.x 14656310 PMC2548277

[B65] Wandell B. A. Dumoulin S. O. Brewer A. A. (2007). Visual field maps in human cortex. *Neuron* 56 366–383. 10.1016/j.neuron.2007.10.012 17964252

[B66] Wang C. Ong J. L. Patanaik A. Zhou J. Chee M. W. (2016). Spontaneous eyelid closures link vigilance fluctuation with fMRI dynamic connectivity states. *Proc. Natl. Acad. Sci. U.S.A.* 113 9653–9658. 10.1073/pnas.1523980113 27512040 PMC5003283

[B67] Wang M. Cui J. Liu Y. Zhou Y. Wang H. Wang Y. (2019). Structural and functional abnormalities of vision-related brain regions in cirrhotic patients: A MRI study. *Neuroradiology* 61 695–702. 10.1007/s00234-019-02199-9 30949745 PMC6511351

[B68] Weissenborn K. (2019). Hepatic encephalopathy: Definition, clinical grading and diagnostic principles. *Drugs* 79 (Suppl. 1), 5–9. 10.1007/s40265-018-1018-z 30706420 PMC6416238

[B69] Weissenborn K. Bokemeyer M. Ahl B. Fischer-Wasels D. Giewekemeyer K. van den Hoff J. (2004). Functional imaging of the brain in patients with liver cirrhosis. *Metab. Brain Dis.* 19 269–280. 10.1023/b:mebr.0000043976.17500.8e 15554422

[B70] Wen M. Yang Z. Wei Y. Huang H. Zheng R. Wang W. (2021). More than just statics: Temporal dynamic changes of intrinsic brain activity in cigarette smoking. *Addict. Biol.* 26:e13050. 10.1111/adb.13050 34085358

[B71] Woo C. W. Chang L. J. Lindquist M. A. Wager T. D. (2017). Building better biomarkers: Brain models in translational neuroimaging. *Nat. Neurosci.* 20 365–377. 10.1038/nn.4478 28230847 PMC5988350

[B72] Yan C. G. Cheung B. Kelly C. Colcombe S. Craddock R. C. Di Martino A. (2013). A comprehensive assessment of regional variation in the impact of head micromovements on functional connectomics. *Neuroimage* 76 183–201. 10.1016/j.neuroimage.2013.03.004 23499792 PMC3896129

[B73] Yang Y. Zhao R. Zhang F. Bai R. Li S. Cui R. (2022). Dynamic changes of amplitude of low-frequency in systemic lupus erythematosus patients with cognitive impairment. *Front. Neurosci.* 16:929383. 10.3389/fnins.2022.929383 36081656 PMC9447953

[B74] Ye M. Guo Z. Li Z. Lin X. Li J. Jiang G. (2020). Aberrant inter-hemispheric coordination characterizes the progression of minimal hepatic encephalopathy in patients with HBV-related cirrhosis. *Neuroimage Clin.* 25:102175. 10.1016/j.nicl.2020.102175 31954985 PMC6965735

[B75] Zang Y. F. He Y. Zhu C. Z. Cao Q. J. Sui M. Q. Liang M. (2007). Altered baseline brain activity in children with ADHD revealed by resting-state functional MRI. *Brain Dev.* 29 83–91. 10.1016/j.braindev.2006.07.002 16919409

[B76] Zhang G. Cheng Y. Liu B. (2017a). Abnormalities of voxel-based whole-brain functional connectivity patterns predict the progression of hepatic encephalopathy. *Brain Imaging Behav.* 11 784–796. 10.1007/s11682-016-9553-2 27138528

[B77] Zhang G. Cheng Y. Shen W. Liu B. Huang L. Xie S. (2017b). The short-term effect of liver transplantation on the low-frequency fluctuation of brain activity in cirrhotic patients with and without overt hepatic encephalopathy. *Brain Imaging Behav.* 11 1849–1861. 10.1007/s11682-016-9659-6 27917450

[B78] Zhang G. Li Y. Zhang X. Huang L. Cheng Y. Shen W. (2020). Identifying mild hepatic encephalopathy based on multi-layer modular algorithm and machine learning. *Front. Neurosci.* 14:627062. 10.3389/fnins.2020.627062 33505243 PMC7829502

[B79] Zhang Y. Wang X. Li Y. (2021). Disrupted dynamic pattern of regional neural activity in early-stage cognitively normal Parkinson’s disease. *Acta Radiol.* 63 1669–1677. 10.1177/02841851211055401 34775837

[B80] Zheng R. Chen Y. Jiang Y. Wen M. Zhou B. Li S. (2021). Dynamic altered amplitude of low-frequency fluctuations in patients with major depressive disorder. *Front. Psychiatry* 12:683610. 10.3389/fpsyt.2021.683610 34349681 PMC8328277

[B81] Zhong W. J. Zhou Z. M. Zhao J. N. Wu W. Guo D. J. (2016). Abnormal spontaneous brain activity in minimal hepatic encephalopathy: Resting-state fMRI study. *Diagn. Interv. Radiol.* 22 196–200. 10.5152/dir.2015.15208 26742646 PMC4790075

