# Supplementary material for: Changes in dynamic and static brain fluctuation distinguish minimal hepatic encephalopathy and cirrhosis patients and predict the severity of liver damage
Source: Front Neurosci. 2023 Mar 28;17:1077808. doi: 10.3389/fnins.2023.1077808 (PMC10086246; doi:10.3389/fnins.2023.1077808)
Supplement: Supplementary file 1 [file Data_Sheet_1.docx]

**Supplementary Material For**

“More than just statics: Changes in static and dynamic brain fluctuation in minimal hepatic encephalopathy with cognitive dysfunction”

Jiang Ji^1,2^, Yi-yang Liu^3^, Guo-Wei Wu^4^, Yan-Long Hu^2^, Chang-Hua Liang^2*^, Xiao-dong Wang^1*^

^1^ Department of Radiology, General Hospital of Ningxia Medical University, Yinchuan, China.

^2^ Department of Radiology, The First Affiliated Hospital of Xinxiang Medical College, Xinxiang, China.

^3^ Department of Radiology, The First Affiliated Hospital of Zhengzhou University, Zhengzhou, China.

^4^Chinese Institute for Brain Research, Beijing, China.

**Supplementary Material 1**
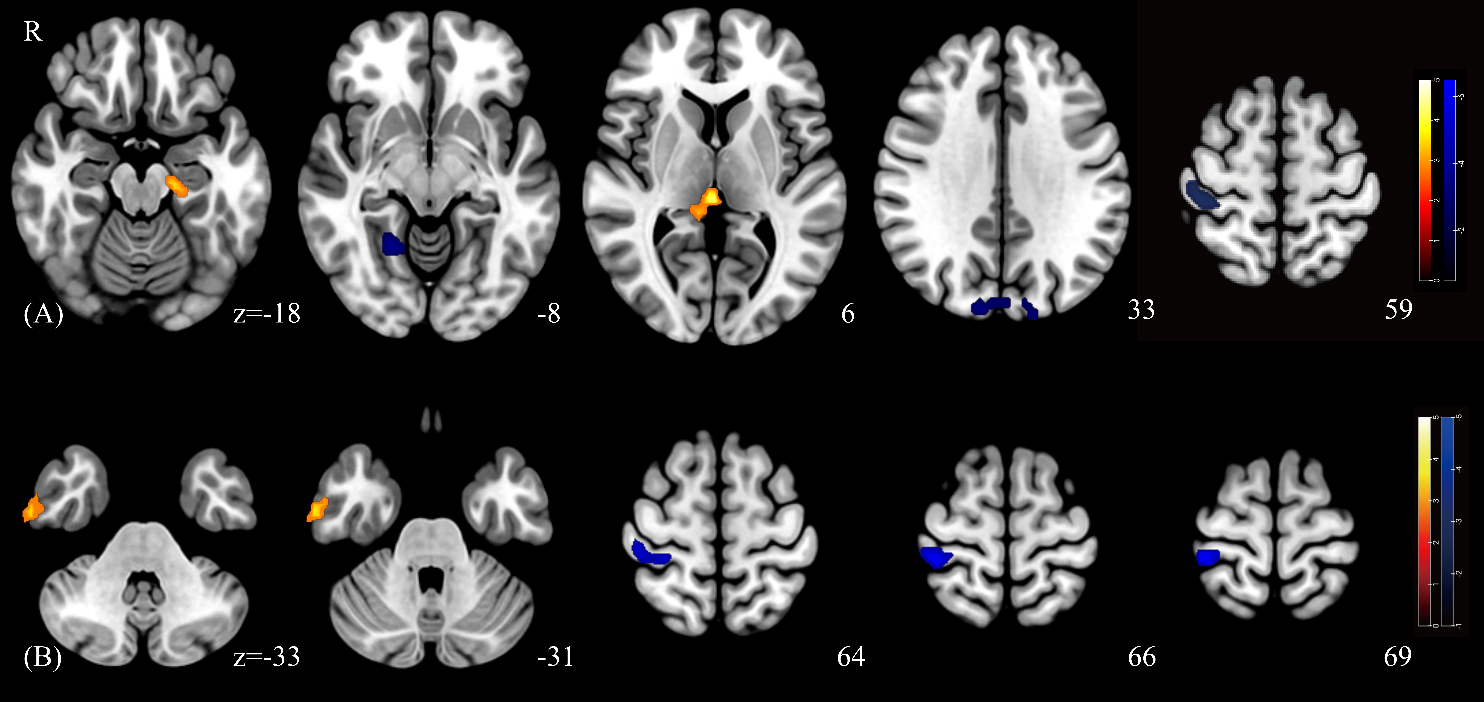


Figure S1 Group differences of temporal variability of intrinsic brain activity. (A) Temporal variability of the sALFF between the MHE and HC groups was identified using two-sample t tests. (B) Significance of the sALFF between the MHE and noMHE groups. The statistical significance level was set at P_voxel_ < 0.005, P_cluster_ < 0.05 (GRF corrected, cluster extent threshold at k ≥ 40). Hot colors represented increased dALFF, and blue colors represented decreased dALFF.

|  | | **TABLE S1 \|** Group differences in ALFF between MHE patients, noMHE patients and HCs. | | | | | | |
| --- | --- | --- | --- | --- | --- | --- | --- | --- |
| Group | Brain regions | | Brodmann area | MNI coordinates | | | Voxels | *T* value |
|  |  | |  | *x* | *y* | *z* |  |  |
| MHE-HC | Left lingual gyrus gyrus | | 30 | -15 | -33 | -12 | 63 | -4.24 |
|  | Bilateral superior occipital gyrus | | 19 | ±12 | -90 | 39 | 74 | -5.04 |
|  | Right postcentral gyrus | | 3 | 45 | -27 | 63 | 83 | -4.42 |
|  | Left parahippocampal gyrus gyrus | | 30 | -15 | -33 | -12 | 63 | 4.24 |
|  | Right thalamus | | - | 3 | -27 | 9 | 74 | 4.76 |
| MHE-noMHE | Right postcentral gyrus | | 3 | 39 | -36 | 69 | 70 | -4.02 |
|  | Right inferior temporal gyrus | | 20 | 46 | 12 | -41 | 46 | 4.62 |
| noMHE-HC | - | | - | - | - | - | - | - |

**Supplementary Material 2**
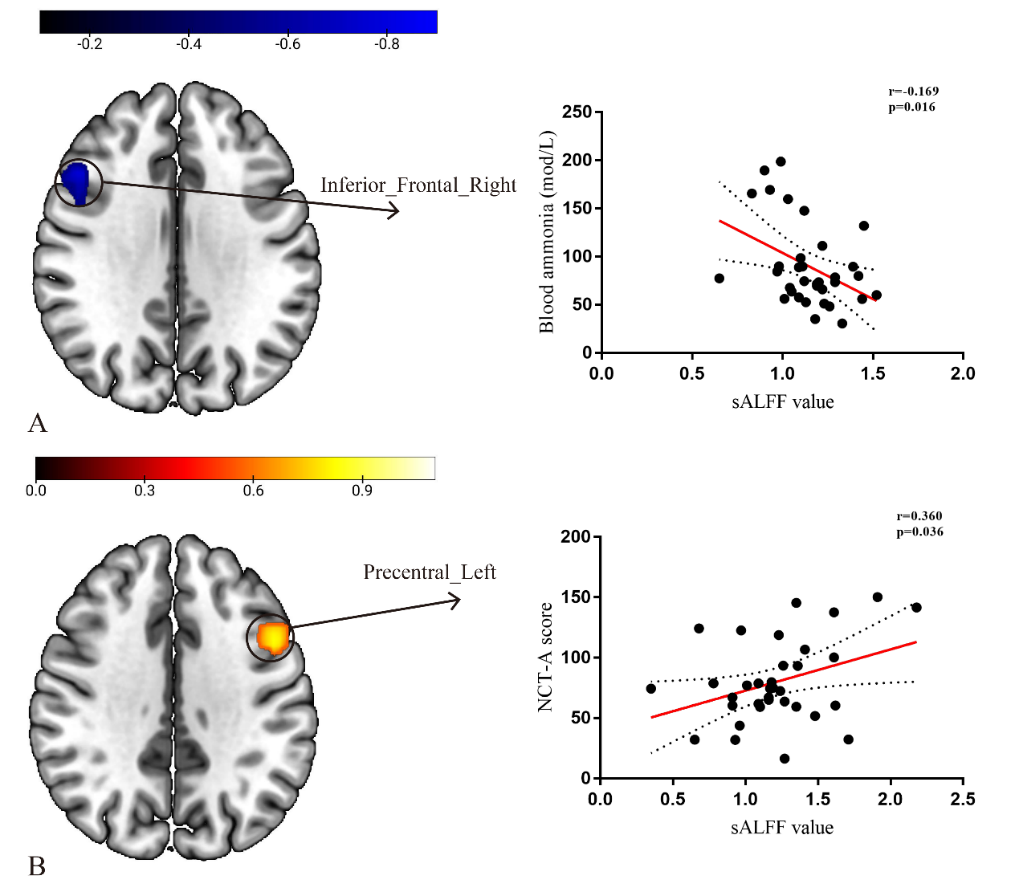


Figure S2 Scatter plot diagram of the correlation between sALFF and blood ammonia, cognition score. (a) The temporal variability of dALFF value in the right inferior frontal gyrus was negatively correlated with blood ammonia (r = - 0.169, p = 0.016, uncorrected). (B) The temporal variability of sALFF value in the left precentral gyrus was positively correlated with NCT-A score (r = 0.360, p = 0.036, uncorrected).

**Supplementary Material 3**
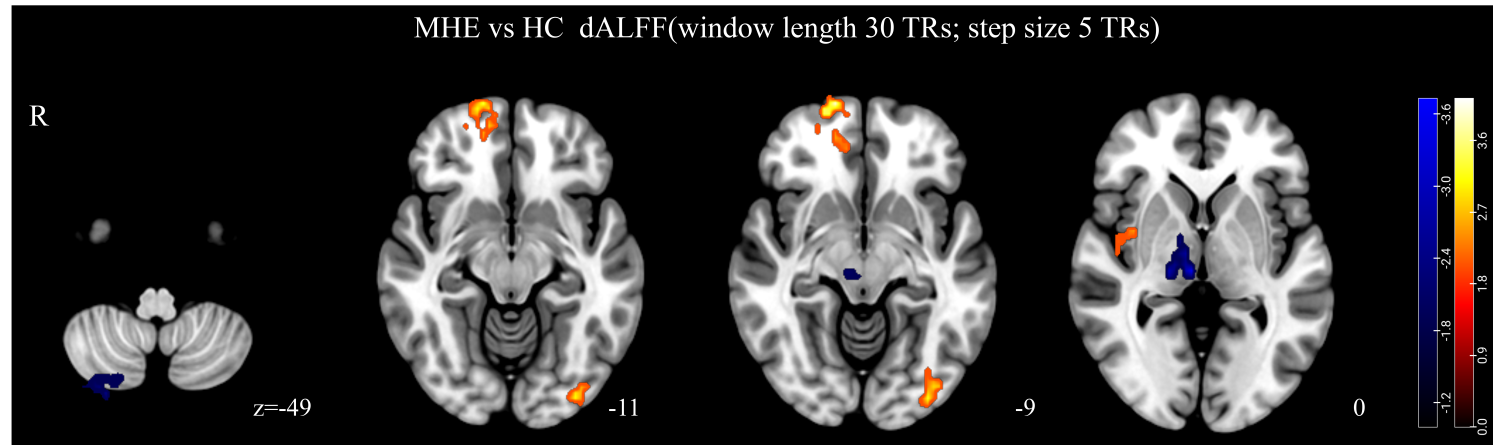


Figure S3a Brain regions with significant group differences in dALFF variability (30 TRs (60s); step size 5 TRs). Significance of the dynamic ALFF between the MHE and HC groups. The statistical significance level was set at P voxel < 0.005, P cluster < 0.05 (GRF corrected, cluster extent threshold at k ≥ 30). Patients with MHE showed significantly increased dALFF variability in the right inferior frontal gyrus, left inferior occipital gyrus and right insula, and decreased dALFF variability in the right cerebellum posterior lobe, and right thalamus.


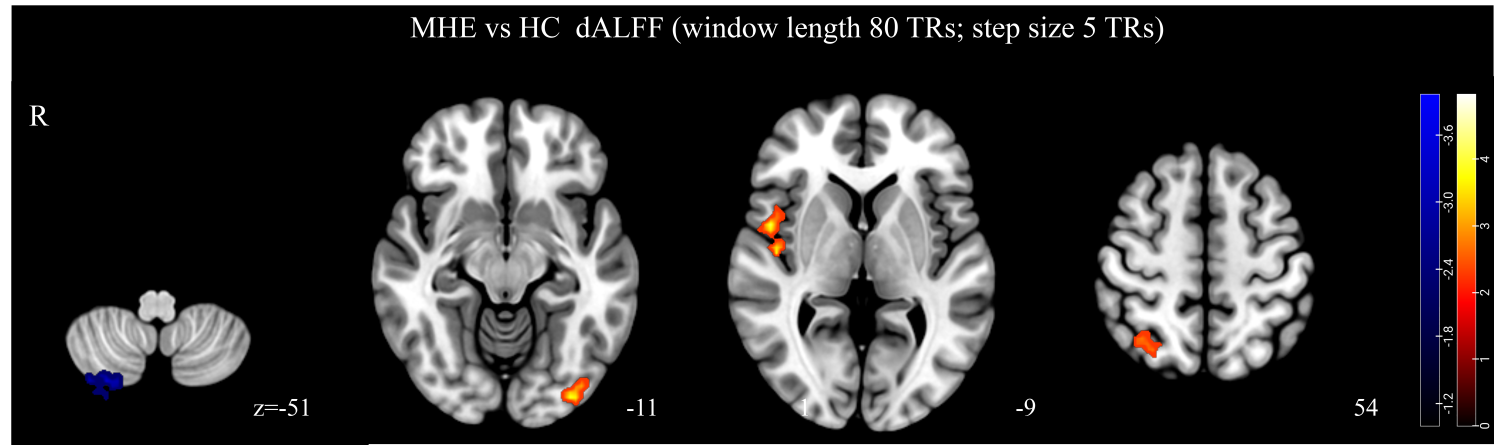


Figure S3b Brain regions with significant group differences in dALFF variability (80 TRs (160s); step size 5 TRs). Significance of the dynamic ALFF between the MHE and HC groups. The statistical significance level was set at P voxel < 0.005, P cluster < 0.05 (GRF corrected, cluster extent threshold at k ≥ 30). Patients with MHE showed significantly increased dALFF variability in the right superior occipital gyrus, left inferior occipital gyrus and right insula, and decreased dALFF variability in the right cerebellum posterior lobe


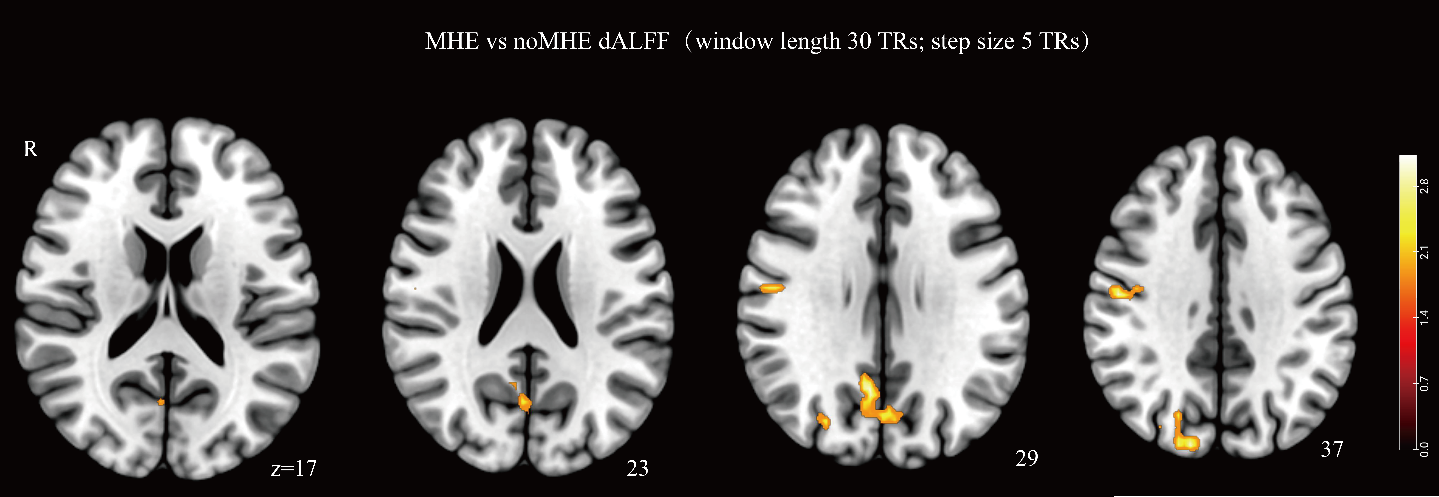


Figure S3c Brain regions with significant group differences in dALFF variability (30 TRs (160s); step size 5 TRs). Significance of the dynamic ALFF between the MHE and noMHE groups. The statistical significance level was set at P voxel < 0.005, P cluster < 0.05 (GRF corrected, cluster extent threshold at k ≥ 30). Patients with MHE showed significantly increased dALFF variability in the right superior occipital gyrus, right superior frontal gyrus and right precuneus.


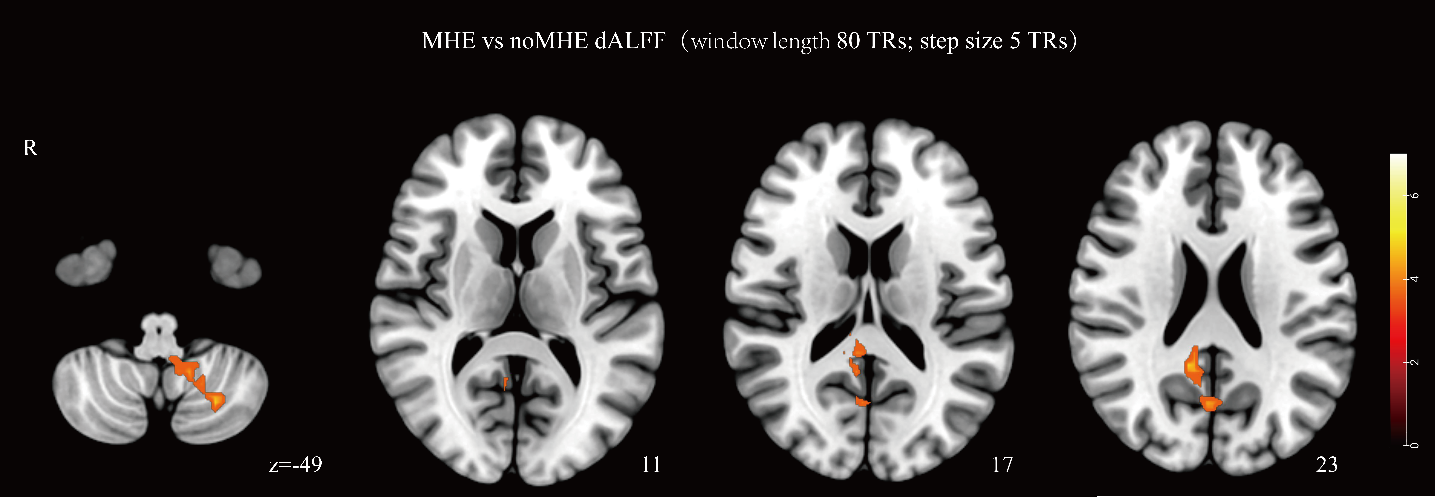


Figure S3d Brain regions with significant group differences in dALFF variability (80 TRs (160s); step size 5 TRs). Significance of the dynamic ALFF between the MHE and noMHE groups. The statistical significance level was set at P voxel < 0.005, P cluster < 0.05 (GRF corrected, cluster extent threshold at k ≥ 30). Patients with MHE showed significantly increased dALFF variability in the right precuneus and left posterior cerebellar lobe.

**Supplementary Material 4**


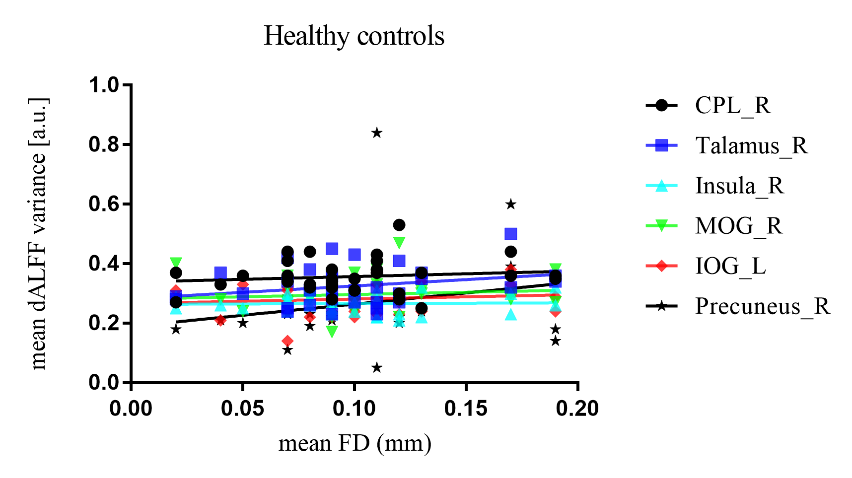

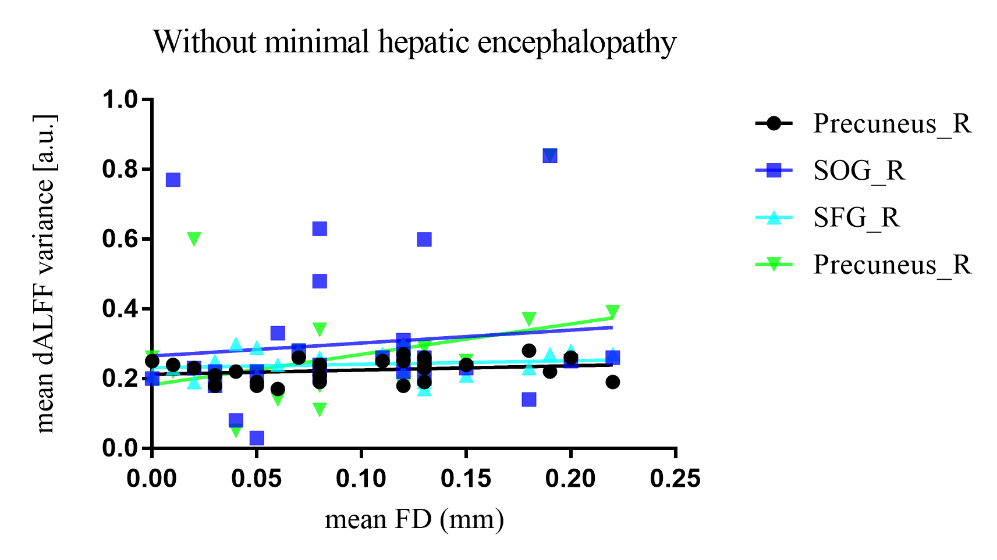

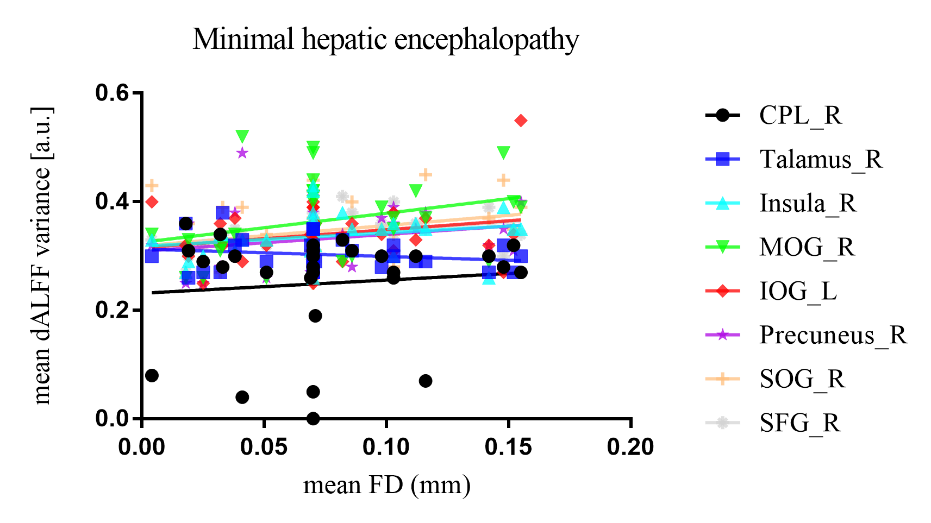


Figure S4 Across subject correlations between mean FD and temporal variability of dALFF from group difference regions. None of the correlation coefficient was significant (all p > 0.05). A, B and C represented correlation analysis in healthy controls, noMHE and MHE, respectively. Filled symbols denote that the data points were included in correlation analyses. Open symbols denote that the data points were outliers. dALFF, dynamic amplitude low-frequency fluctuation; FD, framewise-displacement; CPL, cerebellar posterior lobe; IOG, inferior occipital gyrus; MOG, middle occipital gyrus; SOG, superior occipital gyrus; SFG, superior frontal gyrus.
